# Post SABCS local therapy and radiology

**DOI:** 10.1007/s12254-018-0403-3

**Published:** 2018-05-15

**Authors:** Florian Fitzal

**Affiliations:** Department of Surgery and Breast Health Center, Medical University Vienna, Waehringer Guertel 18–20, 1090 Vienna, Austria

**Keywords:** Breast surgery, Magnetic resonance imaging, Ductal carcinoma in situ, Local therapy, Resection margin

## Abstract

This year there were three interesting oral presentations and several posters presenting important new data regarding local therapy (surgery and radiotherapy) as well as radiological aspects. This minireview is a personal view of the clinically most relevant data in this respect with the following conclusions: A micrometastasis is no indication for axillary dissection. The number of involved sentinel lymph nodes predicts non-sentinel lymph node metastasis and should be taken into account regarding omitting axillary dissection. Neoadjuvant chemotherapy reduces the risk of non-sentinel lymph node metastasis. A 2 mm margin shows an optimal rate of local recurrences after breast conservation. The question of the correct definition for an R0 resection after neoadjuvant therapy remains open. We should omit radiotherapy for women with low risk ductal carcinoma in situ (DCIS) below 2.5 cm in size and pT1a G1 after breast conservation. Risk of finding invasive cancer after having a B3 biopsy is very low depending on the type of lesion, thus, questioning the surgical approach of some of these entities. The use of magnetic resonance imaging is a standard procedure before and after neoadjuvant therapy. Data regarding correlation between complete radiologic response (rCR) with pathologic complete response (pCR) and real tumor size are rare. For women with micrometastases or isolated tumor cells in the sentinel node postmastectomy radiotherapy has little benefit. After neoadjuvant therapy only women with ypN2 had a significant benefit of postmastectomy radiotherapy for local, disease-free and overall survival.

## Omitting axillary dissection

### Micrometastasis and axillary surgery

Vivana Galimberti had an oral presentation about the prospective trial IBCSG 23-01: axillary lymph node dissection versus sentinel only in patients with micrometastases in the sentinel, 10 year data. A total of 931 patients with micrometastases in the sentinel lymph node were included. There were no significant differences comparing axillary dissection with sentinel biopsy regarding disease-free survival events (25% versus 22%), local recurrence (3% both) or regional recurrence (0.6% versus 2%) as shown in Table 1. Interestingly Galimberti also showed data for mastectomized patients (*n* = 86) and patients receiving breast conservation showing no difference with or without axillary dissection regarding ipsilateral axillary events in these two groups. Thus, axillary dissection in patients with micrometastases in the sentinel lymph node and breast conservation is not necessary. These data also strongly suggest that this is true for patients after mastectomy.

**Table 1:** Oncologic outcome comparing axillary dissection with sentinel lymph node biopsy only in patients with micrometastases in the sentinel lymph node

	SNB *N* = 467	ALND *N* = 464
DFS event	101 (22%)	117 (25%)
Local recurrence	14 (3%)	13 (3%)
Regional recurrence	9 (2%)	3 (0.6%)
Distant recurrence	41 (9%)	47 (10%)

*SNB* sentinel lymph nodes biopsy, *ALND* axillary lymph node dissection, *DFS* disease free survival

### Involved nodes and axillary surgery

Vikhepatil showed data from 5694 patients having at least one macro- or micrometastasis between 2008 and 2012 analyzed from their Swedish cancer database. The authors evaluated the risk of non-sentinel lymph node metastases according to the number of positive sentinel lymph nodes. There was a correlation in the number of sentinel lymph node metastases and non-sentinel metastases (non-SNB metastases in 35, 49 and 66% comparing 1SNB+, 2SNB+ or 3SNB+).

### Neoadjuvant therapy and axillary surgery

The first analysis of the French prospective SERC trial comparing sentinel only with axillary dissection in sentinel lymph node metastasized women demonstrated that the risk of non-sentinel lymph node metastases in patients with sentinel lymph node macrometastasis was different comparing high-risk women after neoadjuvant chemotherapy (9%) and women with adjuvant chemotherapy (29%). Low-risk women without any type of chemotherapy had the lowest risk (7%) of non-sentinel lymph node macrometastases. This suggests that we may also think about further trials omitting axillary dissection in patients with sentinel metastasis after neoadjuvant therapy, especially in estrogen receptor negative patients as those have the lowest risk of two or more axillary metastases after neoadjuvant therapy (poster by Namura). In any case all women have to be radiological nodal negative after neoadjuvant chemotherapy.

## Resection margin definition

### Resection margin and local recurrence

Vicini presented their meta-analysis including 38 trials with 55,302 women regarding resection margin and risk of local recurrence. This analysis was different from the large analysis by Houssami as Vicini et al. included 5 more trials and presented updated data from 2 other trials with a total of 20,000 more women. The statistical analysis was very complex and also criticized by Monica Morrow during the discussion. Vicini et al. showed that negative margins in general had significantly fewer local recurrences compared to positive margins. When comparing different resection margins in mm (<1 with >1, <2 with >2, <5 versus >5), all groups were significantly different with a HR of 0.43 to 0.53. The largest significant difference was found between <2 and >2 mm. However the crude rates of 5‑year local recurrence rate in patients with negative margins comparing 1, 2 and 5 mm resection margins were similar (3.5, 3.3 and 3.2%). During the discussion the author concluded that the optimal margin for a low local recurrence rate is 2 mm; however re-resection should be done according to current guidelines (tumor on ink). With a median re-resection rate of 41% in these trials I would strongly challenge the accuracy of mm description. As long as there are no prospective comparative trials we should stick to the definition “no tumor on ink”.

### R0 after neoadjuvant therapy

Choi et al. showed a poster about resection margin after neoadjuvant therapy from 382 women who underwent surgery at the Dana Farber Cancer Center. Interestingly there was no significant difference between close margins (<2 mm, 6%), patients with pathologic complete resection (= pCR; 1%) and negative margins >2 mm (3%). These data are in line with our own analysis from the Medical University Vienna (unpublished data). Data suggest that breast conservation is safe in patients with pCR and worse in patients with residual disease and close margins; however due to the low number of patients there were no statistical differences. Larger meta-analyses are warranted to draw conclusions.

## Precancerous lesions

### Radiotherapy and low risk

Wärnberg prospectively validated a multigenomic biological signature within the SweDCIS trial predicting efficacy of radiotherapy (RT) after surgery. Women diagnosed with ductal carcinoma in situ (DCIS) and treated with breast conservation ± RT were stratified into clinically relevant low and elevated risk groups (Swedish DCIS risk score ≤3 vs >3). Women in the elevated risk group had twice the treatment benefit from RT (HR 0.24), while the low risk group had no benefit from RT (HR 0.83; Table 2).

**Table 2:** The 10-year radiotherapy (RT) benefit in women from the SweDCIS trial

**DS risk groups**		**IBC events**		**In situ or IBC events**	
	***n***	**Absolute RT-benefit** **(%)**	**HR [95%CI]**	Absolute RT-benefit (%)	HR [95%CI]
Low risk group (DS ≤ 3)	243	1	0.83 [0.32–2.16]	9	0.48 [0.24–0.97]
Elevated risk group (DS > 3)	263	9	0.24 [0.08–0.73]	17	0.31 [0.17–0.59]

*DS* DCIS score, *IBC* invasive breast cancer, *RT* radiotherapy

Lalani evaluated 3262 women with DCIS and evaluate the impact of tumor size on recurrence risk in a population of women with pure DCIS treated by breast conservation alone or with radiotherapy (RT). Median follow-up was 13 years (IQR 8‑15 years). On multivariable analyses adjusted for age and year of diagnosis, tumor size ≥40 mm was significantly associated with an increased risk of local recurrence (LR) compared to size ≤10 mm (HR = 2.5, 95%CI: 1.64–3.81). There was a significant interaction between tumor size ≥ 40 mm and RT (*p* = 0.02) while the effect of RT on 15-year invasive local recurrence in smaller lesions was low (15a invasive local recurrence rate <10 mm 11% without RT and 10% with RT; 11–25 mm: 17% without RT and 9% with RT).

Jayasekera showed data from the NSABP. The pooled observational data included women who had undergone breast conserving surgery for stage I, ER+ and/or PR+/HER2− cancers with clinicopathologically estimated 21-Gene Recurrence Scores (RS) of ≤18, and did not receive chemotherapy (*n* = 1684). The 10-year invasive and non-invasive recurrence-free interval was 96.6% with radiotherapy and 91.6% without (absolute difference of 5%). Omission of radiotherapy (vs. radiotherapy) was associated with an overall adjusted hazard ratio of 2.6 (95% confidence interval 1.5–4.5) for any first event. There was only a significant increase in risk of locoregional but not distant recurrence, breast cancer-specific or overall survival. The effect of radiotherapy varied across subgroups, with lower first event rates for those with estimated Oncotype RS of <11 (vs. 11–18), and women ages 60+ (vs. <60 years).

### B3 lesions and cancer risk

Pathologic B3 breast lesions are usually excised by surgical therapy. A recent analysis showed that around 15% of these lesions were malignant and 80% of these were in situ lesions. The risk of finding malignant cells was associated with the subtype of the B3 lesions. According to B3 histologic type breast cancer rates were 12.9% for flat epithelial atypia (FEA), 20% for atypical ductal hyperplasia (ADH), 11.6% for lobular neoplasia (LN), 3.7% for radial scare and 8.8% for papillary lesions.

## Using MRI for pCR and tumor size prognosis after neoadjuvant therapy

### Radiologic correlation after neoadjuvant therapy

Gampenrieder et al. analyzed 246 patients and correlated the pCR with rCR using MRI at a single center. They found that 74% of all pCR patients were correctly assessed; however in 52% the rCR diagnosis was wrong and 26% of the real pCR were overseen by MRI. In general these data showed only a 48% correspondence of rCR to pCR and the author concluded that MRI is not a good clinical tool to clearly detect pCR. The predictive value of MRI for pCR was best in triple negative patients (63%) but still unsatisfying for any treatment decision based on MRI.

A small retrospective analysis of 195 patients was presented by Boersma. All patients operated for breast cancer after NAC between January 2013 and July 2016 in a large teaching hospital were retrospectively included. The longest residual tumor diameter was determined with MRI and correlated with postoperative pathological findings. A total of 193 patients with 195 breast cancers were included. The correlation between tumor size at MRI and pathology was 0.63 for the whole group, 0.39 for tumors with subtype ER+/HER2−, 0.55 for ER+/HER2+, 0.63 for ER−/HER2+ and 0.85 for ER−/HER2−. The correlation for lobular carcinomas was 0.44. The mean difference and limits of agreement (LoA) between tumor size measured by MRI and pathological size was 4.6 mm (LoA −27.0 to 36.3 mm, *n* = 195). The correlation and agreement between the post-NAC MRI and postoperative pathological assessment of residual tumor size for ER+/Her2− and lobular tumors was weak in this small analysis.

## Postmastectomy radiotherapy (PMRT) indication

### Pmrt and lymph node status

Xia et al. identified patients with isolated tumor cells or axillary micrometastases after mastectomy from the Surveillance, Epidemiology, and End Results database from 2004–2014. Overall survival (OS) and breast cancer-specific mortality (BCSM) were compared among patients after PMRT or not using propensity score-matched analyses. From 11,622 eligible cases, PMRT was administered to 1728 patients. From the PMRT group, 1728 (100%) were matched with 1728 patients who did not undergo PMRT. In the matched dataset, OS at 5 years and 10 years were 88.1 and 74.2% in the PMRT group, and were 87.8 and 77.3% in the no PMRT group, respectively. The 5‑year and 10-year cumulative BCSM rates were 6.4 and 12.3% in the PMRT group, and 6.6 and 14.1% in the no PMRT group, respectively. OS and BCSM were unaffected by PMRT after adjusting for multiple confounders (OS, hazard ratio, 0.92; 95% CI, 0.74–1.16; BCSM, subhazard ratio, 0.89; 95% CI, 0.67–1.18).

### After neoadjuvant therapy only women with ypN2 had a significant benefit of postmastectomy radiotherapy for local, disease-free and overall survival

Postmastectomy radiotherapy (PMRT) has been shown to be beneficial in node-positive breast cancer patients. However, the role of PMRT for patients receiving modern neoadjuvant chemotherapy (NAC) is controversial. A retrospective data analysis from patients in the Japanese Breast Cancer Registry evaluated the efficacy of radiotherapy for breast cancer patients treated with NAC and mastectomy. Patients who received NAC and mastectomy for cT1–4 cN0-2 M0 breast cancer were included in this analysis. Of the 145,530 patients registered from 2004–2009, they identified 3226 patients who met the inclusion criteria with the 5‑year follow-up information including 1299 ypN0, 1036 ypN1 and 879 ypN2–3 cases. There was no difference in LRR, DDFS and OS between the groups with and without radiotherapy for ypN1 patients who received NAC (*P* = 0.72, *P* = 0.29 and *P* = 0.36, respectively). For patients with ypN2–3 breast cancer, radiotherapy significantly improved LRR (*P* < 0.001), DDFS (*P* = 0.01) and OS (*P* < 0.001) on univariate analysis. No difference in LRR, DDFS and OS was observed for ypN0 patients (*P* = 0.81, *P* = 0.15 and *P* = 0.05, respectively). In multivariable analysis, the use of radiotherapy was independently associated with improved LRR [hazard ratio (HR): 0.608, 95% confidence interval (CI): 0.452–0.818, *P* = 0.001] and OS [HR: 0.685, 95% CI: 0.531–0.885, *P* = 0.004] for ypN2–3 patients. The results from this nationwide database study of breast cancer patients following modern NAC showed that PMRT did not improve survival for patients with ypN1 and ypN0.

